# Mitigated Impact of Provision of Local Foods Combined with Nutrition Education and Counseling on Young Child Nutritional Status in Cambodia

**DOI:** 10.3390/nu10101450

**Published:** 2018-10-06

**Authors:** Lylia Menasria, Sonia Blaney, Barbara Main, Lenin Vong, Vannary Hun, David Raminashvili, Chhorvann Chhea, Lucie Chiasson, Caroline P. Leblanc

**Affiliations:** 1 École des sciences des aliments, de nutrition et d’étude familiale, Université de Moncton, Moncton, NB E1A 3E9, Canada; lyliamenas@gmail.com (L.M.); caroline.p.leblanc@umoncton.ca (C.P.L.); 2 International Programs, World Vision Canada, 1 World Drive, Mississauga, ON L5T 2Y4, Canada; Barbara_main@worldvision.ca; 3 Independent consultant, Phnom Penh 12203, Cambodia; leninvong17@gmail.com; 4 World Vision Cambodia, Phnom Penh 12203, Cambodia; Vannary_Hun@wvi.org (V.H.); David_Raminashvili@wvi.org (D.R.); 5 National Institute of Public Health (NIPH), Ministry of Health, Phnom Penh 12203, Cambodia; cchhorvann@niph.org.kh; 6 Direction du mieux-être, Ministère du développement social, Miramichi, NB E1N 1B6, Canada; lucie.chiasson@gnb.ca

**Keywords:** Malnutrition, quality complementary foods, young children, nutrition education, nutrition counseling, Cambodia

## Abstract

Background: In Cambodia, stunting and wasting affect, respectively, 32% and 10% of children 0–59 months while 55% are anemic. Our research aims to assess the efficiency of two local foods combined with nutritional education and counseling (CEN) activities as compared to CEN alone on improving child nutritional status and dietary intake. Methods: A cluster-randomized controlled trial was conducted in Soth Nikum area over a six-month period among children 6–23 months (*n* = 360) assigned to receive either moringa +CEN, cricket +CEN or CEN alone. Anthropometric measurements were performed and hemoglobin and ferritin levels assessed. Results: Overall, no significant increase in the mean length/height-for-age *z*-score was observed, although a small increase of the weight-for-length/height was noted in intervention groups. Hemoglobin and ferritin mean values increased in all groups. The degree of satisfaction of energy, proteins, iron, and zinc requirements improved in all groups, but to a greater extent in the intervention groups and more children were healthy. Conclusion: Our research shows no significant impact of the provision of two local foods combined with CEN on the improvement of child nutritional status as compared to CEN alone. However, children consuming them better fulfilled their energy, iron, and zinc requirements and were healthier.

## 1. Introduction

In spite of the progress observed in the last few decades, child malnutrition remains a global concern and a priority [1,2,3]. Worldwide, stunting, and wasting affect 155 and 52 million children below five years of age, respectively, while iron deficiency is the most common and widespread micronutrient disorder [4,5]. Iron deficiency is estimated to contribute around 40% of cases of anemia in children below five years of age [6,7].

Stunting or low length/height-for-age has been associated with increased risk of mortality from infectious diseases, poor child motor and cognitive development, lower educational achievements, reduced physical capacity, and incomes in adulthood as well as increased risks of non-communicable diseases [8]. Anemia has been linked to maternal mortality, low birth weight, delayed child development, and has been used as an indirect indicator of iron deficiency [7]. Studies have also shown that most of the decline in child nutritional status occurs between six and 24 months [9,10]. Thus, supporting the need to make nutrition investments during the 1000 days from conception up to the child’s second birthday for optimal child development [11,12].

Appropriate complementary feeding practices are crucial in the first years of the child’s life to reduce and to prevent malnutrition including iron deficiency anemia [8,13]. The global strategy for infant and young child feeding [14] recommends that, at six months of age, infants receive safe and nutritionally adequate, solid, semi-solid, and soft complementary foods, and that breastfeeding should be continued for up to two years of age and beyond.

Several strategies have been implemented to improve child complementary feeding practices including the intake of iron-rich foods. Behavior change communication such as nutrition education and counseling have been recommended in past years. In food insecure settings, the addition of foods supplements has also been suggested [15,16]. Recently, the results of a systematic review and a meta-analysis showed that in both food secure and insecure settings, complementary feeding interventions (nutrition education or counseling combined or not with food and nutritional supplements) have small but significant impacts on linear and ponderal growth of children aged six–23 months, whereas nutrition education or counseling interventions have an impact on linear growth only [16]. Moreover, nutrition education or counseling had no effect on the length-for-age *z*-score in food-insecure populations, probably because of the limited access to foods. While few studies reported limited results on weight-for-length, no significant impact of nutrition education or counseling intervention was noted on this indicator [16]. Yet, when combined to nutritional supplement, the effect on weight-for-length *z*-score appears to be limited.

According to Lassi et al. (2013), the scarcity of available studies and their heterogeneity as well as the variety in complementary feeding interventions make it difficult to draw conclusions as to their comparative effectiveness [17]. Studies using World Health Organization (WHO) growth standards for the assessment of impacts on nutritional status are also limited as is research conducted in food-insecure settings [16]. Panjwani and Heidkamp (2017) also pointed out that more evidence is needed on the impact of nutrition education interventions on child wasting [16].

In Cambodia, stunting and wasting affect was, respectively, 32% and 10% of children below five years of age [18]. Among young children, micronutrient deficiencies, especially iron and zinc deficiencies, appear to be problematic as approximately 55% are anemic [18,19], while about 70% have low zinc levels [18]. It has also been suggested that micronutrient interventions to improve anemia may have a limited impact on the reduction of anemia prevalence due the fact that more than 40% of anemia is not attributed to nutritional factors [19]. In particular, the high prevalence of genetic hemoglobin disorders may contribute to the elevated prevalence of anemia [19,20]. However, appropriate complementary feeding practices remain limited in Cambodia: Only 36% of children six–23 months benefit from the minimum recommended diet [18]. While not recent and limited in terms of representativeness (around 50 children), two studies [21,22] conducted in five provinces of Cambodia have shown that energy and iron requirements were not satisfied in more than 90% of children six–23 months [21]. Moreover, experience with the “Positive Deviance Hearth” approach has shown that inexpensive, locally available nutritious foods such as frogs, crickets, moringa (*Moringa oleifera*), and some fish species are known but under-utilized by communities [22]. Other efforts to improve nutritional status of Cambodian young children have been undertaken in the past but results showed limited impact [23,24]. Currently, to address the situation, the government of Cambodia is promoting the use of local foods through behavior change communication interventions [25].

To support Cambodia’s efforts to reduce malnutrition, the main objective of this study was to assess the efficiency of moringa and cricket powders (two local foods) combined with nutritional education and counseling activities as compared to nutrition education and counseling alone on the improvement of child nutritional status and dietary intake.

In this study, the selected nutrient-rich foods were moringa and cricket powders given their availability in the area. Both foods were deemed acceptable prior to this research. Recently, insects have received a lot of attention because of their potential to improve the nutrition status of populations [26]. In Cambodia, insects such as crickets are part of the local population diet. For instance, in the Siem Reap area, crickets can be purchased in local public markets. Although moringa is a plant in which minerals (such as iron, zinc) absorption may not be optimal, given its availability and consumption by Cambodian populations (as a soup or integrated into local recipes such as Samlor Karko), and because of the limited research on the impact of this plant-based food on nutritional status, it was selected as one of the supplementary foods in this trial.

## 2. Materials and Methods

### 2.1. Theoretical Framework

The research is based on the United Nations Children’s Fund (UNICEF) conceptual framework on the causes of malnutrition, which considers dietary intake and health status as the immediate determinants of child nutritional status [27]. These, in turn, depend on underlying determinants: access to adequate food, appropriate care, adequate health services, and a healthy environment. In order for each determinant to be fulfilled, resources should be available at the community level but also reach households and each member [28].

### 2.2. Preparatory Work

Before undertaking the trial, local enumerators (*n* = 38) were trained on data collection methodology including anthropometry measurements. Community health workers (2 per selected village) were also trained on nutrition counseling and education using national training packages developed by the National Nutrition programme from the National Maternal and Child Health Center, Ministry of Health. All survey tools were translated in Khmer and reviewed during the training to ensure their conformity with the English version as well as the absence of any ambiguity. A pretest of all survey tools was also conducted in villages located in the vicinity of the training center, which was far from villages selected for the study. 

### 2.3. Study Design and Sample

This cluster-randomized controlled community trial was conducted in Soth Nikum Operation District of Siem Reap province over a six-month period. The area is the intervention zone of an international non-governmental organization (NGO), which was looking to initiate a new nutrition program. The site is located near the renowned Angkor Wat temples. The province has been classified as chronically food-insecure [29], with high rates of stunting (36%) among children under five [15]. The sample size was defined to detect a statistically significant improvement in child nutritional status measured by a mean improvement of 1 g/dL of hemoglobin level among each group of children between baseline and at 6 months of study (endline) implementation assuming, 90% power and a 5% significance level [30]. Based on available data, the mean hemoglobin level among children 6–23 months was at 10.1 ± 1.3 g/dL [18]. The hemoglobin level was chosen for the sample size calculation because the aim of our intervention was to improve the overall child nutritional status, and in particular, iron status given the high prevalence of anemia and its association with iron deficiency. Among populations, anemia is generally assessed through the measurement of hemoglobin concentration [7]. Therefore, we recruited a total of 360 children who were equally assigned to one of the three groups (120 per group, accounting for 10% attrition). Specifically, to define the sample, all children aged 6–23 months in all villages of the area were first listed and 14 villages were randomly selected and assigned to one of the following treatments: a) supplementary food #1 (moringa powder) plus nutrition education and counselling, b) supplementary food #2 (cricket powder) plus nutrition education and counselling, and c) nutrition education and counselling (CEN). For each cluster, 4–5 villages were thus selected so as to obtain 120 children 6–23 months per cluster. Villages of different clusters were far from each other to reduce contamination or spillover among groups.

Both moringa and crickets were powdered locally. Moringa powder was produced by Baca Villa production Co. Ltd (Siem Reap, Cambodia). Cricket powder was made by a local vendor (Pursat, Cambodia) trained on food safety issues for cricket. Tests were performed by a Canadian laboratory to ensure relative safety of both foods. Foods were packaged into 16 g (moringa) and 41 g (cricket) hermetically sealed sachets. Selected foods were distributed on a weekly basis to participating households by NGO project staff. The distribution was initiated after the completion of the baseline study. Each child received a daily ration of 16 g of moringa and 41 g of cricket powder so as to provide around 1.5 mg of iron daily, which corresponds to around 20% of the daily requirements [31]. Moringa powder contains 388 kcal per 100 g, 33 g of protein, 10 mg of iron, 1.4 mg of zinc and 356 mg of vitamin C. Cricket powder provided 478 kcal/100 g, 56 g of protein, 6.1–6.6 mg of iron, 17–18 mg of zinc and no vitamin C. Energy and nutrient composition of moringa (two samples) was assessed by Tuv Rheinlan Co Ltd, (Phnom Penh, Cambodia), while the cricket powder (eight samples) was analyzed by the agriculture and food laboratory of Guelph University, Guelph, ON, Canada, and detailed nutrition content was provided by Maxxam Analytics Int. Corp., Mississauga, ON, Canada. 

In addition to the provision of moringa and cricket powder, caregivers were invited to participate in two group sessions of nutrition education on a monthly basis. Every caregiver was also offered a counseling (face-to-face) session on a monthly basis. The emphasis of nutrition education was on child feeding including the importance of time, appropriate complementary foods including nutrient-rich foods, and meal frequency, responsive feeding, hand washing, food consistency, food preparation. Group and counseling sessions were provided by trained community health workers.

Mothers and children 6–23 months in each selected village were invited to participate on a voluntary basis to the survey and free to cease participation at any moment. Exclusion criteria included children with severe anemia or malnutrition, children from families which may not be present in the area for the entire study period, children from multiple births and those with congenital anomalies. In the study area, there was no other provision of food supplements or multiple micronutrient powders.

### 2.4. Data Collection

Data on nutritional status and its determinants as well as on socioeconomics were collected through baseline and endline surveys. At midterm, the data collection was simplified. Between surveys, home visits (three times per week) were conducted to promote the consumption of moringa and cricket, as well as caring practices. 

#### 2.4.1. Anthropometric and Biochemical Nutrition Data, Faeces Collection

At baseline, midterm and endline surveys, children and their mother/caregiver were weighed with an electronic scale (UNISCALE, 150 ± 0.1 kg) by two trained enumerators following the recommended procedure [32]. For children aged 24 months or more, height was measured vertically while length of children below 24 months was taken in a lying position with a standardized board (UNICEF, 130 ± 0.1 cm). For each child, the date of birth was collected from the health card, other official document, or caregiver’s report. 

A trained team of health staff from the capital city collected venous blood (3–4 mL) into two tubes. The first one contained the anticoagulant (ethylenediaminetetraacetic acid/EDTA) (Siem Reap, Cambodia) for hemoglobin testing. Blood (3–4 mL) for C-Reactive Protein (CRP) (Siem Reap, Cambodia) and ferritin testing was collected in a second tube. All specimens were kept in triple packaging in a cool box containing ice-packs and then sent to the National Institute of Public Health laboratory in Phnom Penh by taxi (around 5 h). Children presenting severe anemia were excluded and referred for treatment to the nearest health facility.

A stool sample was collected from each child during the household surveys at baseline and endline. As such, caregivers were given a container with formalin to preserve the sample and child identification number and instructions on how to collect and store the sample. The container was collected from the caregiver and transported to the laboratory.

#### 2.4.2. Dietary Intake

At baseline, midterm and endline surveys, data on child food intake was collected through three (3) quantified 24-h recalls/survey carried out during two different days of the week and one weekend day. The frequency of breastfeeding over each previous 24 h was also recorded. Food quantities were estimated using local utensils. Information on vitamins and minerals supplements and food supplementation was also verified. 

#### 2.4.3. Health Status, Health and Feeding Practices, Household Food Security, Access to Health and Socioeconomics

Semi-structured interviews were conducted at baseline and endline in a quiet place with (i) the head of each household together with caregivers and (ii) privately with the latter and wife or caregiver of the targeted child in the household. During the first interview, socio-demographics data were collected on each household (e.g., age, sex, schooling, access to a healthy environment and improved water source, housing condition, asset ownership, child’s birth weight) and household food security. The second interview aimed to gather data on health and feeding practices including child health status. The Demographic and Health Survey (DHS) 2014 Cambodia questionnaire was adapted to collect the aforementioned data [18]. Household food security status was assessed using version three of Food and Nutrition Technical Assistance’s (FANTA) Household Food Insecurity Access Scale (HFIAS) measurement tool, which has been pretested and adapted for use in Cambodia [33,34].

### 2.5. Data Analysis

#### 2.5.1. Nutritional Status

For children under five years of age, length/height-for-age (stunting) and weight-for-length/height (wasting) indices were calculated using the WHO Anthro 2011 software (version 3.2.2, Geneva, Switzerland). Children with indices below -2 *z*-scores from the median reference values of the WHO growth standards were considered undernourished. In caregivers, a body mass index (BMI) below 18.5 and over/equal to 25 indicates underweight and overweight respectively [32].

Hemoglobin was tested using the fluorescence flow cytometry technology (model XT-1800i, Sysmex machine, (Norderstedt, Germany) while ferritin was analyzed using Cobas e601 5 Analyzer Roche Diagnostics, (Indianapolis, IN, USA). In regard to iron status, a hemoglobin level below 11 g/dL indicates anemia among children while a value below 12 µg/L highlights limited/no iron storage for ferritin [31]. Iron status among children was assessed by combining results of hemoglobin (<11 g/dL) and ferritin (12 µg/L) levels. As ferritin is elevated in the presence of inflammation, young children with an elevated CRP (>5 mg/L) were excluded from the analysis [5].

Child birth weight (BW) was classified in one of two categories namely (1) Low BW: if <2.5 kg and, (2) Adequate BW: if ≥2.5 kg. 

#### 2.5.2. Food and Nutrient Intake

The consumption of cricket and moringa was assessed from three recalls conducted at midterm and endline in addition to those administrated at baseline. Each child was classified as follows, according to his consumption of cricket and moringa both, at midterm and endline.

Data on child dietary intake were analyzed with the Nutrific software (Laval University, Quebec, Quebec, Canada) to calculate the average daily energy and nutrient intake (proteins, iron, zinc and vitamin C). The nutritive value of foods not originally included in the software was added to the database from information on labels or from other sources such as the Asian food composition database and the Sustainable Micronutrient Interventions to Control Deficiencies and Improve Nutritional Status and General Health in Asia (SMILING) user food composition table for Cambodia [35]. All data from recalls were entered first by research assistants and a graduate student and thereafter, checked a second time by another research assistant and the main author to ensure the quality of the data entry.

#### 2.5.3. Satisfaction of Nutrient Requirements

For each child, nutrient requirements were estimated from the WHO/Food and Agriculture Organization of the United Nations (FAO) recommendations for energy, macro and micronutrients [31,36,37]. For iron requirements, a diet of 10% of iron bioavailability was considered while for zinc requirements, a diet with moderate zinc bioavailability was used. The degree of satisfaction (%) of energy and nutrient requirements was calculated by comparing the daily child intakes (including that from breastmilk) with the estimated requirements based on age and breastfeeding status [38]. Breastfed children were classified according to the frequency of breastfeeding during the 24-h period as follows: (1) low: 1 to 4 times, (2) average: 5–14 times, (3) high: ≥14 times/day. Categories were defined based on the mean daily average and the standard deviation. Using these categories, on a daily basis, energy and nutrients from breastmilk were derived for each child and added to intakes from other foods and liquids consumed during the 24-h period. 

#### 2.5.4. Health Status

To assess health status, three indicators were considered. First, for each child, a score on health status was developed based on whether or not she/he was presenting signs of illnesses during the past 14 days preceding the survey. A score of three (3) was assigned to children who had diarrhea, acute respiratory infection and fever in the past two weeks preceding the survey, two (2) if he had only two illnesses, and one (1), one illness. A score of zero was assigned if he had none of these illnesses. Moreover, the CRP value was considered as an indicator of health status as well as the presence of intestinal parasites. The CRP was tested using the immunoturbometric method (model 7180, Bayer machine, (Leverkusen, Germany). The presence of intestinal parasite infestation was examined microscopically using the formalin-ether sedimentation technique [39].

#### 2.5.5. Household Socioeconomic Level

A factor analysis using principal axis factoring was performed on the correlation matrix to define a socioeconomic score for each household. Initially, 18 items on ownership of assets and housing conditions were used but the final score considered 11 items (ownership of a fan, television, wardrobe, motorcycle and bicycle, watch, boat, mobile phone, and radio having electricity and number of rooms in the house), which explains 29, 90% of the total variance and composition of the first factor. The Kaiser-Meyer-Olkin Test, which assesses the suitability of the data for the factor analysis was 0.803 which is satisfactory [40].

### 2.6. Statistical Tests

Anthropometric food and nutrient intakes data were transferred to SPSS (Version 21.0, IBM Corporation, Armonk, NY, USA) for further analysis. All other data were entered directly into SPSS. 

Normality of the distribution pattern was continuous variables was examined by visual inspection of the probability plots and with the Kolmogorov-Smirnov test. Homogeneity of the variance was assessed with the Levene test. When necessary, data transformations (e.g., log) were applied to obtain normal distribution of the data and homoscedasticity.

For continuously distributed variables, the Generalized Linear Modeling (GLM) procedure for repeated measures was used to assess differences between baseline, midterm, and endline. The Greenhouse-Geisser value was checked to detect overall significant differences between data of each survey. The Student Newman-Keuls post hoc test was used to identify which specific means differed. For categorical variables, the Cochran Q and the chi-squared tests were used to assess differences in their distribution. For all analyses, a probability value of 0.05 was considered as significant.

### 2.7. Ethical Approval

The research protocol was approved by the National Ethics Committee for Health Research, Ministry of Health, Cambodia (367-NECHR) and the Ethic committee on human research of the Université de Moncton, Moncton, New Brunswick, Canada (#1617-037). Verbal and written consents from all participants were also obtained. Survey description was also read to each participant and they were all informed that they could leave the study whenever they wanted.

## 3. Results

Overall, there were no differences in sociodemographic characteristics between groups at baseline and endline (Table 1). However, at endline, the proportion of children born with a low birth weight was more elevated among children of the MgE group, as compared to MgO. At baseline, the mean socioeconomic score was higher in the control group as compared to cricket and moringa groups.

At midterm, 78% of all children assigned to the moringa group were consuming the powder for three days and 66% of those assigned to the cricket group were eating cricket (results not shown). All of them were consuming the powder for at least one day out of three. Moreover, 60% and 7% of children consumed more than 50% of the daily ration of moringa (16 g) and cricket (41 g) powders at midterm, respectively. At endline, less than 10% (9% for moringa and 1% of cricket) of children were consuming the food for three days. However, 100% and 79% of those children were eating more than 50% of the daily ration of moringa and cricket. In addition, 25% and 58% ate moringa and cricket, respectively, for at least one day out of three, while 66% and 41% did not consume moringa and cricket, respectively, at all (results not shown).

### 3.1. Nutritional Status

Table 2 shows results on nutritional status indicators in all groups at baseline, midterm and endline. Overall, there was no difference in the length/height-for-age mean *z*-score (L/HAZ) in the CME group between baseline, midterm and endline. However, in this group, the weight-for-length/height mean *z*-score (WL/HZ) improved between midterm and endline, but there was no difference between baseline and endline. A decline between baseline and endline was observed in the CMO group for L/HAZ but not for WL/HZ which remained unchanged throughout the study. At endline, children of the CMO group had a lower L/HAZ as compared to CME and control groups. There were no differences among the three groups with regard to other nutrition indicators at endline. Similarly, there were also no differences in L/HAZ, WL/HZ or prevalence of stunting and wasting between the control group and CME or CMO at baseline and midterm. 

Between baseline, midterm and endline, the overall prevalence of stunting increased in the cricket group (from 20.7% at baseline to 26.9% and 42.3% at midterm and endline, respectively) but remained unchanged in the moringa (17.4%, 11.4%, 23.0%) and control (19.3%, 10.9%, 22.9%) groups (results not shown, *p* = 0.000). In the moringa and cricket groups, no differences in prevalence of wasting and WL/HZ were observed between baseline and endline (results not shown). 

In the CkE and CkO groups, L/HAZ decreased between baseline and endline but no difference was noted between baseline and midterm. There was also no difference for the WL/HZ throughout the study period in these two groups. In the MgE group, no significant difference was observed with regard to L/HAZ as well as the WL/HZ between baseline, midterm and endline. However, in the MgO group, a decline of the WL/HZ was noted between baseline and midterm but no change was observed between midterm and endline (Table 2). 

In the control group, L/HAZ and WL/HZ did not change between baseline and endline. However, L/HAZ decreased from midterm to endline while WL/HZ decreased between baseline and midterm. Lastly, between baseline and endline, levels of hemoglobin and ferritin improved in all groups including the control group. However, the overall proportion of children with both, a low hemoglobin and ferritin levels, remained stable between baseline (19.4%, *n* = 56/288) and endline (23.6%, *n* = 63/267), results not shown.

### 3.2. Consumption of Cricket or Moringa

The number of children consuming cricket and moringa went down from 82 to 22, and from 95 to 56, respectively, between midterm and endline (Table 3). The average number of days of cricket and moringa consumption also decreased from 2.7 and 3.2 days to 0.4 and 1.1 days respectively between midterm and endline. However, mean daily quantities were higher at endline for cricket and CMO groups. 

### 3.3. Satisfaction of Energy and Nutrient Requirements

At baseline, the degree of satisfaction of proteins requirements was higher in the CME as compared to CMO and the control group (Table 4). The degree of satisfaction of iron requirements was also higher in the CME and control groups as compared to the CMO group. At midterm and endline, the degree of satisfaction of energy, proteins, iron and zinc requirements was generally higher in the CME group, as compared to other groups. 

Overall, the degree of satisfaction of energy requirements among children in CME and CMO groups increased from baseline to midterm, and then decreased at endline, while it increased for zinc especially between midterm and endline. The decline in the degree of satisfaction of energy requirements between midterm and endline was higher in the CMO while in CME group, the endline level was the same as that at baseline. In both groups, the degree of satisfaction of proteins and iron requirements increased between baseline and midterm, but stagnated between midterm and endline (Table 4). Specifically, in CkE and MgE groups, the degree of satisfaction of nutrient requirements usually increased from baseline to midterm and was constant between midterm and endline with the exception of the degree of satisfaction of zinc requirements that still improved between midterm and endline. In the CkO group, there was an increase of the degree of satisfaction of energy, protein and iron requirements between baseline and midterm but no change between midterm and endline with the exception of energy requirements, which decreased. In the MgO group, with the exception of iron, no significant change in the satisfaction of requirements was observed between baseline, midterm, and endline. With the exception of the CkE group, the degree of satisfaction of vitamin C did not change between baseline, midterm, and endline in all groups (Table 4).

In the control group, the degree of satisfaction of energy requirements increased between baseline and midterm, but decreased at endline. The degree of satisfaction of other nutrient requirements remained similar between baseline and midterm, but significant increases in the degree of satisfaction of protein, iron, and zinc requirements were observed between baseline and endline.

### 3.4. Health Status 

At baseline, no differences were noted in the proportion of healthy children for all groups (19.7% to 26.5%). Overall, proportions of healthy children significantly increased from baseline, midterm and endline in each group (Table 5). Yet, at midterm and endline, the proportion of healthy children was significantly lower in the control group at 53.3%, as compared to CME group at 70.1% and CMO group at 78.8%. Between baseline and endline, no differences were observed between proportions of children with intestinal parasites or for those with an elevated CRP value.

## 4. Discussion

We assessed the efficacy of consuming moringa and cricket powders combined with CEN among young children and compared it with CEN alone on the improvement of young child nutritional status and dietary intake. Our hypothesis was that a higher improvement would occur in the first two groups as compared to the group benefiting from CEN only. 

Overall, adding supplementary foods to CEN activities did not improve young child nutritional status, as compared to CEN alone. Between baseline and the end of the six-month period (endline) of food supplementation, there were no changes in the nutritional status among children considered compliant to the intervention, namely the CME group. Also, no change was observed in the control group between baseline and endline. However, there was an improvement of WL/HZ between baseline and midterm in the CME group. In the CMO group, where children were consuming cricket or moringa either at midterm or endline, there was a decline of the L/HAZ between baseline and endline. Moreover, no change was observed with regard to the L/HAZ and WL/HZ in the MgE group while no clear conclusion can be drawn from the CkE group, given the small sample size. In general, a decline of young child nutritional status was noted in the CkO and MgO groups between baseline and endline. In all groups, between baseline and endline, there was an improvement of indicators related to iron status in all groups, but no differences between groups at any point of time. 

It appears that nutrition education and counseling alone was as much as effective as combining it with food supplements, with regard to the impact on child anthropometry: No improvement was observed in the CME, CMO, and the control groups between baseline and endline or between groups in general at any point of time. However, at midterm, the improvement of WL/HZ in the CME group may be attributed to the consumption of cricket or moringa, which were eaten by a higher number of children and over at least three days a week. Perhaps if the consumption would have been sustained until the end of the six-month period, an impact on child nutritional status would have been observed. Yet, there was no deterioration of nutritional status among children of CME and control groups as compared to the general population of young children in Cambodia [18]. Moreover, in all groups, iron status improved between baseline and endline, but no difference was observed among groups at endline.

The lack of impact of the provision of food supplements combined with CEN, as compared to CEN alone on child nutritional status may be attributed to several causes. First, the contribution of cricket and moringa to the satisfaction of energy and nutrient requirements, and thus, to the improvement of nutritional status was certainly limited given than around 20 g and 8 g of each food was consumed daily, which represents about an additional 95 and 30 kcal per day. Second, given the high promotion of cricket and moringa by local health agents, in the case of child’s refusal or inadequate food preparation, caregivers may have limited other (complementary) foods. Third, it is likely that more changes in nutritional status could have occurred in the absence of intestinal parasite infections. However, all children were affected with intestinal parasites to the same extent at endline and one can suppose that the situation was similar in all groups throughout the study. Still, at baseline, to ensure optimal impact of the consumption of cricket and moringa on child nutritional status, all children affected by intestinal parasites were treated as per the national guidelines. They were likely infested again thereafter, as shown by the absence of differences between proportions of children with intestinal parasites between baseline and endline. With regard to iron status as measured by hemoglobin and ferritin levels, their improvement may be due to the child’s aging. In Cambodia, Reinbott et al. (2016) have shown a steady increase of hemoglobin and ferritin levels among young children between 9–10 and 22–24 months of age [41]. Therefore, our sample size may have been defined based on a mean increase of hemoglobin level, which is naturally occurring within a young child population during the first years of their life. Yet, our sample size was also sufficient to detect a mean increase of 0.5 *z*-score for the L/HAZ in each group between baseline and endline, assuming 80% power and a 5% significance level [25]. In addition, our measurements of iron status may not have been optimal. While it has been recommended to use serum ferritin in addition to hemoglobin to assess if anemia is caused by iron deficiency [7], elevated ferritin levels were also observed in the presence of hemoglobin disorders, which constitutes a limitation for its use to assess iron deficiency anemia in our population [20].

Our results are in line with previous studies. In a recent systematic review and meta-analysis, Panjwani and Heidkamp (2017) found no effect of nutrition education or counseling alone on LAZ score in food-insecure populations [16]. Similar to our study, they also report no significant impact of CEN alone on the improvement of weight-for-length. Moreover, in food-insecure settings, when complementary food supplement was combined or not with nutrition education, the effect on LAZ and WLZ score was small. On the contrary, the results of a previous systematic review conducted by Lassi et al. (2013) show that education on complementary feeding alone improved HAZ and WAZ scores among young children from in a food insecure population [17]. However, this review included only one study which assessed the impact of education on HAZ score in a food insecure setting such ours. According to Imdad et al. (2011), the impact of nutrition education depends on educational messages and availability of foods [42]. Educational interventions had more impact when there is an emphasis on feeding nutrient-rich animal-source foods. In our case, although nutrition messages were delivered on young child feeding to caregivers on a monthly basis, they were not specifically insisting on feeding animal-source foods. 

Our results are also consistent with the efficacy trial conducted by Bauserman et al. (2015) in DRC, which did not show any impact of the provision of caterpillar cereal combined with nutrition education on stunting and anemia reduction among children six–18 months even though the cereal provided an extra of 132 kcal and 3.8 mg of iron daily and was supplied to children during a 12-month period [43]. In a study carried out in India among children four–12 months of age aiming to compare the impact of provision of a milk cereal supplement combined with encouragements to feed and monthly group counseling over an eight-month period, no impact on wasting and stunting reduction was noted [44]. In Cambodia, the impact of nutrition education program aiming to improve infant and young child feeding behaviors combined with agricultural interventions did demonstrate improved diet quality among children under two years of age, yet no impact on nutritional status was observed [45].

The decline of L/HAZ in the CMO group is unexpected. At the bare minimum, we anticipated no change in young child nutritional status since similarly to the control group, the CMO group benefited from CEN activities. Besides, at midterm or endline, some children of the CMO group were also consuming cricket or moringa. The higher proportion of healthy children in cricket and moringa groups compared to the control group, despite no improvement in nutritional status is of interest. It is possible that the additional, but small quantity of nutrients from cricket and moringa were used to fight illness instead of growth.

Our study shows an overall improvement in the degree of satisfaction of energy and nutrient requirements at midterm in CME and CMO groups, including CkE, MgE, CkO, and MgO groups with the exception of vitamin C. Furthermore, the satisfaction of energy and nutrient requirements was generally higher in the CME group at midterm and endline, as compared to CMO and control groups. This might be attributed to the consumption of cricket and moringa. In spite of the fact that small quantities were consumed at both times, both foods were still an extra source of energy and nutrients for young children. The decrease or stagnation of the degree of satisfaction of energy and nutrients requirements observed between midterm and endline is likely due to the reduction of the food consumption in terms of the number of children consuming each food as well as the frequency of consumption. It is possible that there was a fatigue associated with the long-term consumption of cricket and moringa among children. This is clearly demonstrated by our data on cricket and moringa consumption, which shows a decrease of number of days of consumption as well as of numbers of children consuming cricket and moringa between midterm and endline. Our results also show that CEN alone contributed to the improvement in the degree of satisfaction of energy, protein and iron requirements especially between baseline and midterm. In India, Bhandari et al. (2001) also observed higher energy intakes among children whose caregivers attended nutrition counseling in group sessions on a monthly basis during an eight-month period [44].

To our knowledge, no other study has provided accurate data on young child dietary intake and satisfaction of their energy and nutrient requirements in Cambodia. Even though cricket and moringa were provided to children, our results show that energy, iron, zinc, and vitamin C intakes were inadequate while protein intake exceeded requirements. It is possible that children do not eat enough, which can result in low energy and nutrient intakes. Food consistency may also be an issue: Young children are often given watery porridges/foods. With regards to protein, even though intakes were superior to requirements, protein quality may not be optimal. In fact, cereal-based diets may provide less than adequate amounts of protein requirements, and one can question the impact of suboptimal protein quality on child growth [46]. In this population, it has also been suggested that micronutrient interventions to improve anemia may have a limited impact on the reduction of anemia prevalence due the fact that more than 40% of anemia is not attributed to nutritional factors [19]. Given that only a quarter of young children appear to fulfill their iron requirements and around 20% have low iron status, one should not exclude intervention such as CEN and even micronutrient supplementation to improve iron status. Thus, our results support the conclusions of Wieringa et al. (2016) to broaden the iron intervention to zinc supplementation and deworming measures [19].

Our study has several limitations. First, there was a reduction in total number of children consuming cricket and moringa from midterm to endline as well as a decline in the number of days of consumption. In fact, not all children consumed suggested amount of foods on a daily basis. This situation could have limited the power to detect significant differences between groups. The attrition was most likely attributed to fatigue in consuming both foods. In addition, the quantities of foods offered were small which may have restrained their potential to make substantial changes on child nutritional status. Therefore, a better understanding of potential barriers to the consumption of moringa and cricket should have been acquired beforehand and addressed during CEN interventions to enhance adherence to these foods. One other major limitation to our trial is the absence of a control group who did not benefit from any intervention, but this option was not ethically acceptable. Nutrition education and counseling activities may also have varied in terms of quality among different groups and thus, it could have affected child dietary intake differently across all groups. We suppose that it may have been happening in some groups and not only in one. In addition to the aforementioned limitations, at baseline, children included in the CME were also better off with regard to the satisfaction of protein requirements as compared to CMO and the control group while the degree of satisfaction of iron requirements was lower in the CMO group, as compared to the CME and control groups. This situation was accentuated at midterm and endline. However, there were still improvements in the degree of satisfaction of energy and zinc in the CME group. While some caution should be taken when comparing groups with each other especially with regards to the improvement of protein requirements, as there were improvements of dietary intake among the CME group at midterm, most likely because of the consumption of cricket and moringa. Given the high probability of genetic hemoglobin disorders among young Cambodian children, which has been documented [18,20], it would have also been useful to assess the prevalence of this problem among our population to get a better understanding of causes of anemia. Finally, because of the nature of the design of our study (a cluster-randomized controlled trial), there might be other factors that could have influenced the impact of our interventions and were not considered in our research. For instance, the sanitation and hygiene situation may have been better in the control group at baseline given its higher socioeconomic status, which may explain the absence of impact of our interventions on the young child nutritional status between the control and the CME groups. 

In spite of limitations, our research provides valuable information regarding young child dietary intake. To our knowledge, no research has provided accurate data on the situation of young child dietary intake in Cambodia neither reported on the efficacy of local foods for improving child nutrition. Moreover, it also reports outcomes on WL/HZ using the WHO growth standards that have been rarely addressed by previous research [16]. While local foods such as moringa and cricket have not shown a significant impact of child nutritional status, they have likely contributed to the improvement of dietary intake and health status. 

## 5. Conclusions

In Siem Reap area, child malnutrition is a public health concern. Our research shows no significant impact of the provision of two local foods, moringa, and cricket combined with nutrition education and counselling on the improvement of child nutritional status as compared to CEN alone. Yet, there was no deterioration of nutritional status among children of the control group as it will be generally observed in the overall population of young children. Moreover, children consuming moringa and cricket fulfilled, to a greater extent, their energy, iron, and zinc requirements as compared to those not eating them. Additionally, greater proportions of children consuming cricket and moringa were healthy as compared to the control group. For future programs, we underline the importance of dedicating more efforts to the provision of quality nutrition education and counseling in food insecure settings including the promotion of the use of local foods such as moringa and cricket to ensure optimal energy and nutrient intakes and improved health among young children. CEN interventions should also further integrate behavior-change communications on disease control such as prevention of parasitic infections so as to maximize their impact on young child nutritional status.

## Figures and Tables

**Table 1:** Description of the population at baseline, midterm and endline *.

	Baseline **			Midterm					Endline				
Characteristics	Ck	Mg	Ct	CkE	CkO	MgE	MgO	Ct	CkE	CkO	MgE	MgO	Ct
Infant characteristics	(129)	(115)	(112)	(82)	(23)	(94)	(10)	(104)	(20)	(82)	(52)	(44)	(101)
% of male	56,2	45,2	45,4	59,8	60,9	47,9	40,0	47,1	50,0	56,1	46,2	43,2	49,5
% with LBW	10,3	5,3	12,3	12,8	10,0	6,9	0,0	12,2	5,0	13,9	11,5 ⱡ	0,0	11,6
Mean age in months (± standard-deviation/SD)	13,7 (4,9)	13,9 (4,9)	14,1 (5,2)	16,8 (4,2)	15,2 (5,3)	16,9 (4,6)	17,2 (5,2)	16,5 (4,9)	20,7 (4,9)	19,9 (5,3)	20,4 (4,7)	20,0 (4,9)	20,1 (5,1)
Caregiver’s characteristics													
None	26,4	29,8	26,4	25,0	38,1	29,5	33,3	25,5	20,0	26,9	35,8	29,5	24,4
1-6 years	51,2	46,5	54,5	48,8	42,9	46,6	22,2	53,9	55,0	52,6	49,1	43,2	50,0
≥7 years	22,3	23,7	19,1	26,3	19,0	23,9	44,4	20,6	25,0	20,5	15,1	27,3	25,5
Mean age (years)	29,9	29,8	30,5	29,6	29,4	30,7	27,1	29,8	27,8	30,6	32,2	28,3	20,2
	(9,1)	(9,4)	(10,2)	(9,1)	(7,7)	(10,3)	(6,0)	(9,4)	(4,8)	(9,8)	(10,0)	(9,4)	(5,1)
Mean BMI	22,2	22,1	22,0	22,4	22,4	22,0	24,5	22,0	22,6	22,2	22,5	21,8	22,0
	(3,5)	(3,5)	(2,8)	(3,9)	(3,9)	(3,4)	(4,0)	(2,8)	(4,0)	(3,5)	(3,3)	(3,6)	(2,8)
Household’s characteristics													
Sex of head (male, %)	96,9	96,7	94,6	96,3	95,7	96,8	90,0	95,2	95,2	97,6	100,0	91,3	95,0
Means size	5,0	4,9	4,9	5,2	4,8	5,1	4,4	4,9	4,4	5,2	5,4	4,6	4,9
	(1,9)	(1,,9)	(2,0)	(2,0)	(1,6)	(1,9)	(1,6)	(1,8)	(1,4)	(1,9)	(2,1)	(1,6)	(1,8)
Socioeconomic score	−0,1 ⱡ (1,0)	−0,1 (0,9)	0,2 (1,1)						−0,2 (0,9)	−0,1 (0,9)	−0,1 (0,9)	−0,3 (0,8)	0,3 (0,8)

* At midterm, 29 and 20 children were absent in the cricket and moringa groups respectively while at endline, numbers of missing children were 18 and 19 respectively. ** Ck: Cricket group (all children), Mg: Moringa (all children); Ct: Control; CkE: Cricket Eaters; MgE: Moringa Eaters; CkO: Cricket Others; MgO: Moringa Others, LBW: low birth weight, BMI: body-mass index. ⱡ Indicate significant differences between groups.

**Table 2:** Nutritional status of sub-groups of the children population involved in baseline, midterm and endline: prevalence of undernutrition (%) and mean *z*-score (± standard deviation/SD) for anthropometric and biochemical indicators *.

Indicator	Baseline							Midterm **							Endline						
	CkE **	CkO	MgE	MgO	CME	CMO	Ct	CkE	CkO	MgE	MgO	CME	CMO	Ct	CkE	CkO	MgE	MgO	CME	CMO	Ct
	(16)	(64)	(40)	(26)	(56)	(90)	(92)	(16)	(64)	(40)	(26)	(56)	(90)	(92)	(16)	(64)	(40)	(26)	(56)	(90)	(92)
Stunting %	26,3 (18)	17,4 ⱡ (103)	9,4 (49)	21,1 (66)	13,9 (67)	18,8 (169)	18,8 (109)	26,3 (17)	17,4 (76)	11,3 (51)	5,6 (37)	15,3 (68)	12,9 (113)	8,9 (92)	47,4 (19)	27,8 (79)	20,8 (45)	12,7 (42)	27,8 (64)	22,0 (120)	19,6 (96)
L/HAZ	−1,46 ^a^	−1,22 ^a^	−1,01	−1,11	−1,14	−1,19 ^a^	−1,06 ^a,c^	−1,40 ^a,b^	−1,24 ^a^	−0,99	−0,63	−1,11	−1,06 ^a^	−0,87 ^a^	−1,82 ^b^	−2,03 ^b^	−1,18	−1,03	−1,36	−1,74 ^b,ⱡ,^^Т^	−1,32 ^b c^
score	(0,91)	(1,08)	(1,20)	(1,00)	(1,13)	(1,05)	(1,31)	(1,28)	(1,38)	(0,94)	(0,99)	(1,05)	(1,30)	(1,22)	(0,88)	(1,25)	(1,08)	(0,86)	(1,06)	(1,23)	(0,94)
Wasting %	10,5 (18)	9,6 (103)	11,3 (49)	5,6 (66)	11,1 (67)	8,1 (169)	5,4 (109)	10,5 (17)	8,7 (76)	11,3 (51)	5,6 (37)	11,1 (68)	7,5 (113)	12,5 (92)	5,3 (19)	16,5 (79)	13,2 (45)	2,8 (42)	11,1 (64)	8,1 (120)	10,7 (96)
WL/HZ	−0,54	−1,03	−0,92	−0,30 ^a c^	−0,81 ^a b^	−0,82	−0,75 ^a^	−1,02	−1,13	−0,99	−0,62 ^b^	−1,00 ^a^	−0,98	−1,15 ^b^	−0,62	−1,16	−0,83	−0,62 ^b c^	−0,77 ^b^	−1,00	−0,95 ^a,b^
score	(0,99)	(0,92)	(0,93)	(0,82)	(0,91)	(0,95)	(1,00)	(1,01)	(0,92)	(0,94)	(1,00)	(0,98)	(0,96)	(0,88)	(0,60)	(0,98)	(1,08)	(0,90)	(0,97)	(0,98)	(0,88)
Hb	9,7 ^a^ (1,0) *n* = 15	9,9 ^a^ (1,3) *n* = 55	10,1 ^a^ (1,1) *n* = 39	10,3 ^a^ (0,9) *n* = 33	10,0 ^a^ (1,1) *n* = 54	10,0 ^a^ (1,2) *n* = 88	9,9 ^a^ (1,1) *n* = 79	-	-	-	-	-	-	-	10,2 ^b^ (0,8) *n* = 15	10,6 ^b^ (1,2) *n* = 55	10,6 ^b^ (1,1) *n* = 39	10,6 ^b^ (1,2) *n* = 33	10,5 ^b^ (1,1) *n* = 54	10,6 ^b^ (1,2) *n* = 89	10,3 ^b^ (1,0) *n* = 83
Ferritin	19,9 ^a^ (14,9) *n* = 15	20,7 ^a^ (17,4) *n* = 56	23,2 (19,2) *n* = 39	20,5 ^a^ (15,6) *n* = 33	22,3 ^a^ (18,0) *n* = 54	20,6 ^a^ (16,6) *n* = 89	23,3 ^a^ (23,4) *n* = 83	-	-	-	-	-	-	-	27,7 ^b^ (20,2) *n* = 15	34,5 ^b^ (33,9) *n* = 55	31,9 (23,8) *n* = 39	33,8 ^b^ (20,1) *n* = 33	30,7 ^b^ (22,8) *n* = 54	34,3 ^b^ (29,4) *n* = 89	32,9 ^b^ (9,7) *n* = 83

* Different letters show significant differences between the same groups at different times for mean scores (baseline, midterm and endline). ** CkE: Cricket Eaters; MgE: Moringa Eaters; CkO: Cricket Others; MgO: Moringa Others; CME: Cricket or Moringa Eaters; CMO: Cricket or Moringa Others; Ct: Control; L/HAZ: Length/height-for-age *z*-score; WL/WHZ: Weight-for-length/height z score; Hb: Hemoglobin. ⱡ Indicate significant differences between proportions for the same group between baseline, midterm or endline. ^Т^ Indicate significant differences between CME, CMO and Ct groups at one point of time.

**Table 3:** Mean (± SD) daily quantity of foods (g) and mean number of days of complementary food consumption among treatment groups at midterm and baseline as measured over a 3-day period *.

Treatment group	Quantity of foods (g)				Number of days			
	Midterm		Endline		Midterm		Endline	
	*N*	Mean	*N*	Mean	*N*	Mean	*N*	Mean
Cricket	82	16,52 ^a^ (10,57)	22	24,19 ^b^ (10,35)	82	2,7 ^a^ (1,1)	22	0,4 ^b^ (0,7)
Moringa	95	8,06 (4,56)	56	8,34 (5,07)	95	3,2 ^a^ (1,1)	56	1,1 ^b^ (1,2)
All (both foods)	177	11,98 ^a^ (8,97)	78	12,81 ^b^ (9,96)	177	3,0 ^a^ (1,1)	78	0,8 ^b^ (1,1)

* Different letters show significant differences between midterm and endline for each group (*p* < 0.05).

**Table 4:** Mean degree of satisfaction (± SD) of nutritional requirements among sub-groups of children at baseline, midterm and endline *.

	Baseline							Midterm							Endline						
	CkE ** (16)	CkO (70)	MgE (50)	MgO (43)	CME (66)	CMO (113)	Ct (96)	CkE (16)	CkO (70)	MgE (50)	MgO (43)	CME (66)	CMO (113)	Ct (96)	CkE (16)	CkO (70)	MgE (50)	MgO (43)	CME (66)	CMO (113)	Ct (96)
Ener	84,8 (29,3)	78,6 ^a^ (23,1)	86,5 ^a^ (30,3)	83,0(31,6)	86,1 ^a^ (29,6)	80,3 ^a^ (26,6)	75,9 ^a^ (26,4)	89,2 (41,7)	98,4 ^b^ (86,6)	117,7 ^b^ (92,6)	96,2 (63,0)	110,8 ^b^ (83,8)	97,6 ^b^ (78,2)	95,6 ^b,^^Т^ (70,7)	71,3 (15,7)	60,7 ^c^ (21,9)	90,5 ^a,c^ (44,3)	80,9 (36,1)	85,8 ^a^ (40,0)	68,4 ^c^ (29,7)	68,8 ^c,^^Т^ (25,5)
Prot.	192,7 ^a^ (66,9)	165,5 ^a^ (79,5)	211,2 (134,1)	172,5 ^a^ (70,8)	206,7 ^a^ (121,1)	168,2 ^a^ (76,0)	179,1 ^a,^^Т^ (74,0)	271,6 ^b^ (89,5)	216,8 ^b^ (92,4)	241,8 (107,0)	225,7 ^a,b^ (204,8)	249,0 ^b^ (103,1)	220,2 ^b^ (145,0)	197,8 ^a,b,^^Т^ (84,7)	252,5 ^b^ (67,6)	228,7 ^b^ (102,0)	258,4 (168,6)	222,0 ^b^ (105,6)	257,0 ^b^ (150,0)	226,2 ^b^ (103,0)	201,2 ^b,^^Т^ (90,7)
Iron	22,7 ^a^ (17,8)	15,6 ^a^ (11,9)	25,0 ^a^ (20,4)	20,0 ^a^ (16,6)	24,4 ^a^ (19,7)	17,3 ^a^ (14,0)	25,5 ^a,^^Т^ (24,4)	37,1 ^b^ (18,9)	30,0 ^b^ (25,5)	55,8 ^b^ (26,6)	39,5 ^b^ (24,5)	51,3 ^b^ (26,1)	33,9 ^b^ (25,4)	31,1 ^a,b,^^Т^ (25,6)	36,5 ^a,b^ (28,3)	28,5 ^b^ (16,9)	59,0 ^b^ (33,0)	35,6 ^b^ (24,7)	53,5 ^b^ (33,2)	31,2 ^b^ (20,4)	33,4 ^b,^^Т^ (22,1)
Zinc	48,1 ^a^ (16,6)	41,9 ^a^ (19,7)	49,9 ^a^ (25,3)	49,5 ^a^ (33,1)	49,4 ^a^ (23,4)	44,8 ^a^ (25,8)	43,2 ^a^ (18,6)	49,7 ^a,b^ (19,5)	42,8 ^a^ (17,5)	60,5 ^b^ (25,6)	49,7 ^a,b^ (24,0)	57,9 ^b^ (24,5)	45,5 ^a^ (20,4)	47,8 ^a,^^Т^ (21,5)	58,1 ^c^ (20,5)	50,0 ^b^ (19,0)	68,1 ^c^ (37,2)	60,8 ^a,c^ (38,9)	65,7 ^c^ (34,0)	54,1 ^b^ (28,5)	49,2 ^b,^^Т^ (18,9)
VitC	108,5 ^a^ (67,3)	78,0 (41,8)	100,7 (64,4)	94,0 (72,1)	102,6 (64,7)	84,1 (55,6)	88,4 (65,6)	67,3 ^b^ (45,0)	74,7 (73,7)	100,4 (60,2)	71,0 (50,3)	92,3 (58,3)	73,3 (65,5)	82,0 (53,4)	57,6 ^b^ (42,7)	66,2 (50,2)	109,0 (70,0)	78,8 (68,5)	96,5 (67,9)	71,0 (57,9)	94,5 ^Т^ (83,6)

* Different letters indicate significant differences (*p* < 0.05) for the same group between baseline, midterm and endline; ** CkE: Cricket Eaters; MgE: Moringa Eaters; CkO: Cricket Others; MgO: Moringa Others; CME: Cricket or Moringa Eaters; CMO: Cricket or Moringa Others; Ct: Control; Ener: Energy; Prot: Proteins. ^Т^ Indicate significant differences between CME, CMO and Ct groups at one point of time.

**Table 5:** Health status among sub-groups of children at baseline, midterm and endline.

Health status indicator	Baseline							Midterm **							Endline						
	CkE **	CkO	MgE	MgO	CME	CMO	Ct	CkE	CkO	MgE	MgO	CME	CMO	Ct	CkE	CkO	MgE	MgO	CME	CMO	Ct
% healthy, past 14 days	22,2 ⱡ (*n* = 18)	24,3 ⱡ (103)	26,5 ⱡ (49)	19,7 ⱡ (66)	25,3 ⱡ (67)	22,4 ⱡ (169)	23,6 ⱡ (110)	36,8 (19)	48,4 (93)	54,7 (53)	76,4 (55)	50,0 (72)	58,8 (148)	34,3 ^Т^ (105)	88,2 (17)	82,3 (79)	64,0 (50)	73,6 (53)	70,1 (67)	78,8 (132)	53,3 ^Т^ (92)
% with intestinal parasites	14,3 (12)	30,9 (56)	20,0 (32)	31,9 (32)	18,5 (44)	31,3 (88)	27,7 (68)	-	-	-	-	-	-	-	33,3 (12)	27,3 (56)	22,4 (38)	17,4 (38)	25,4 (50)	23,6 (94)	22,4 (76)
% with elevated CRP	0 (15)	2,3 (88)	11,4 (44)	7,0 (57)	8,5 (59)	4,1 (145)	6,8 (102)	-	-	-	-	-	-	-	15,8 (19)	1,7 (115)	0 (53)	2,8 (71)	4,2 (72)	2,1 (186)	6,2 (112)

** CkE: Cricket Eaters; MgE: Moringa Eaters; CkO: Cricket Others; MgO: Moringa Others; CME: Cricket or Moringa Eaters; CMO: Cricket or Moringa Others; Ct: Control. ⱡ Indicate significant differences between proportions for the same group at different times (baseline, midterm and endline). ^Т^ Indicate significant differences between CME, CMO and Ct groups at one point of time.

## Abbreviation

**Acronym**	**Definition**
CkO	Cricket consumed 1 of 3 times at midterm OR endline (Cricket Others)
MgO	Moringa consumed 1 of 3 times at midterm OR endline (Moringa Others)
CMO	Cricket or moringa consumed 1 of 3 times at midterm OR endline (Cricket or Moringa Others)
CkE	Cricket consumed 1 of 3 times at midterm AND endline (Cricket Eaters)
MgE	Moringa consumed 1 of 3 times at midterm AND endline (Moringa Eaters)
CME	Cricket or moringa consumed 1 of 3 times at midterm AND endline (Cricket or Moringa Eaters)
